# Suicidal Behavior in University Students in Spain: a Network Analysis

**DOI:** 10.1002/brb3.70457

**Published:** 2025-04-18

**Authors:** Victoria Soto‐Sanz, Álvaro García del Castillo‐López, David Pineda, Raquel Falcó, Tíscar Rodríguez‐Jiménez, Juan C. Marzo, José A. Piqueras

**Affiliations:** ^1^ Miguel Hernández University of Elche Alicante Spain; ^2^ Analysis and Psychological Intervention in the Prevention of Health Risk Behaviours Research Group (PREVENGO) Elche Spain; ^3^ University of La Rioja La Rioja Spain; ^4^ University of Zaragoza Teruel Spain

**Keywords:** emotional well‐being, mental health, network analysis, suicidal behavior, suicidal ideation, suicide, university, youth

## Abstract

**Introduction:**

Suicidal behavior is a significant mental health concern among university students, yet it remains underexplored through a network approach. Traditional methods often overlook the complex interplay of psychological factors influencing suicidal behavior. This study addresses this gap by (a) examining suicidal behavior using network analysis and (b) identifying the psychological network of suicidal behavior along with protective and risk factors among university students.

**Method:**

The sample consisted of a total of 1048 Spanish university students (*M* = 20.5 years; SD = 2.5; 58.2% women) from various universities. Several instruments were used to measure suicidal behavior, emotional and behavioral difficulties, prosocial behavior, subjective well‐being, emotional intelligence, self‐esteem, empathy, affect (both positive and negative), and emotional regulation. Data were analyzed using network analysis to understand the relationships among these variables.

**Results:**

A total of 21.5% of the participants had experienced suicidal thoughts, 26.3% had wished to be dead or not wake up, 15% had considered specific suicide methods or made plans, and 5.8% had attempted suicide at least once. In addition, 18% had engaged in non‐suicidal self‐injury (NSSI) at least once. Network analysis revealed that suicidal behavior was highly interconnected with other psychological factors, with “considered taking own life” as the most influential node. Key factors included depressive symptoms, positive affect, and emotional intelligence. Suicidal behavior showed a positive connection with depressive symptoms and negative affect and a negative connection with self‐esteem and positive affect. Stability analysis confirmed the accuracy of the network estimations, indicating reliable insights into the psychological interconnections.

**Conclusions:**

These findings indicate that suicidal behavior in university students is a complex, dynamic system shaped by cognitive, emotional, and affective factors. Network analysis, through advanced psychopathological and psychometric models, offers valuable insights into suicidal behavior, improving risk and protective factor assessment. This highlights the need for targeted and comprehensive prevention strategies in educational settings.

## Graphical abstract

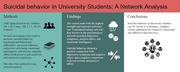

## 1 Introduction

Suicide has become a public health problem worldwide, ranking as the third leading cause of death among individuals aged 15–29 (World Health Organization [WHO] 2024). It represents a significant burden in terms of years of potential life lost and disability‐adjusted life years (WHO 2021). The economic impact is also considerable, with direct and indirect costs of mental health disorders exceeding 4% of the gross domestic product in many countries (OECD, 2021).

Globally, suicide remains a leading cause of death among university students (Centers for Disease Control and Prevention 2023). Among this population, a high prevalence of suicidal ideation, non‐suicidal self‐injury (NSSI), and suicide attempts has been well documented (Auerbach et al. 2018; Eskin et al. 2016; Brausch and Muehlenkamp 2018; Kiekens et al. 2023; Mortier et al. 2018; Saraff and Pepper 2014; Taliaferro and Muehlenkamp 2015; Whitlock et al. 2011). Studies in high‐income countries estimate that 20%–30% of university students experience suicidal ideation, while 5%–10% attempt suicide at some point (Mortier et al. 2018; Twenge et al. 2018). In Spain, 9.9% of university students report suicidal ideation, 5.6% have made suicide plans, and 0.6% have attempted suicide in the past year (Blasco et al. 2019). In addition, NSSI is highly prevalent, with rates ranging from 7.5% to 46.5%, depending on the population studied (Cipriano et al. 2017).

The transition to adulthood is characterized by identity exploration, increased responsibilities, and changing social roles (Arnett 2023). University students experience academic pressure, social expectations, and the challenges of independent living, which contribute to higher levels of psychological distress (Mortier et al. 2018). The COVID‐19 pandemic further exacerbated these difficulties, leading to an increase in anxiety, depression, and sleep disturbances (Calati et al. 2022). Reports indicate that suicidal ideation among students nearly doubled in 2020, primarily due to social isolation, financial insecurity, and academic disruptions (Bersia et al. 2024; Brailovskaia et al. 2021).

Risk factors for suicidal behavior are multifaceted and complex, encompassing biological, psychological, and social dimensions. Among the most studied psychosocial risk factors are depressive symptoms, emotional dysregulation, and social disconnection, which heighten vulnerability to suicide (Twenge et al. 2018). Additionally, stressful life events, particularly interpersonal, chronic, and independent stressors, have been identified as significant contributors to suicidal behavior (López‐Fernández et al. 2024). Problematic social media use and cybervictimization have also been linked to increased suicidal ideation and behavior, as exposure to harmful online content can negatively impact mental health (Marchant et al. 2017).

While research has predominantly focused on high‐income countries, studies in low‐ and middle‐income countries (LMICs) highlight additional risk factors such as limited access to mental health care, economic hardship, and cultural stigma (Musyimi et al. 2020). In Spain, victimization experiences, such as bullying and cybervictimization, have been linked to suicidal behavior, particularly among adolescents exposed to adverse relational contexts (Jiménez et al. 2024). Conversely, family communication quality has been identified as a key protective factor, buffering the negative effects of victimization on suicidal ideation (Buelga et al. 2024).

Despite these risk factors, several protective factors help mitigate suicide risk. Research on suicidal behavior prevention has increasingly focused on positive psychological resources, highlighting emotional intelligence (EI) as a key protective factor (Domínguez‐García and Fernández‐Berrocal 2018). EI plays a crucial role in emotion regulation, adaptive coping strategies, and social connectedness, which are essential in reducing suicidal ideation and behavior (Quintana‐Orts et al. 2020). Studies have shown that individuals with higher EI exhibit lower levels of depression, anxiety, and self‐harm and are more likely to engage in help‐seeking behaviors (Soto‐Sanz et al. 2019). In addition, studies emphasize the role of positive beliefs, self‐esteem, social support, family communication, and coping mechanisms in reducing suicidal ideation and behavior (Buelga et al. 2024; Castellví et al. 2017; Jiménez et al. 2024; Soto‐Sanz et al. 2019). Specifically, reasons to live during suicidal crises have been identified as a key protective factor, reinforcing the need for interventions that promote resilience and adaptive coping strategies (Besch et al. 2024).

Suicide research has shifted from reductionist psychiatric models to holistic, multidimensional frameworks (O'Connor and Nock 2014). Recent studies emphasize the importance of integrating a phenomenological, humanized, and holistic approach to suicide prevention (Al‐Halabí and Fonseca‐Pedrero 2024). The Integrated Motivational‐Volitional (IMV) Model (O'Connor 2011) highlights cognitive, social, and environmental influences on suicidal behavior. Increasingly, contemporary suicide prevention frameworks emphasize meaning–making, resilience, and social determinants—moving beyond purely medical models (Al‐Halabí and Fonseca‐Pedrero 2023). Effective psychotherapies incorporate emotion regulation, cognitive restructuring, and problem‐solving, while comprehensive strategies integrate community interventions and policy initiatives to reduce suicide rates (Hawton and Sinyor 2025).

A growing body of research supports the use of ambulatory assessment methods, which provide real‐time data on fluctuations in suicidal ideation and associated risk factors, improving risk detection and personalized interventions (Fonseca‐Pedrero et al. 2022; Kleiman et al., 2017). These methods align with recent calls for a humanized and holistic perspective, integrating social determinants and lived experiences (Al‐Halabí and Fonseca‐Pedrero 2023, 2024).

Despite identifying numerous risk and protective factors, little is known about how they interact dynamically. Network analysis has emerged as a valuable method for understanding suicidal behavior, conceptualizing symptoms as interconnected rather than isolated variables (Borsboom and Cramer 2013; Robinaugh et al. 2020). This approach has been applied to suicidal ideation in adults and university students (Karnick et al. 2022; Ordóñez‐Carrasco et al. 2023), suicide‐related behaviors linked to adverse life events (Nazir et al. 2014), and psychopathology in clinical populations (Rath et al. 2019). In addition, studies have used network analysis to identify key psychosocial variables in adolescent suicidal behavior (Fonseca‐Pedrero 2018). Despite extensive research on risk and protective factors, little is known about their dynamic interplay, highlighting the need for network‐based approaches to better understand suicide risk (De Beurs 2017).

The present study aims to fill these gaps by analyzing the network structure of suicidal behavior in a sample of university students. Specifically, the study seeks to estimate the psychological network structure between suicidal behavior and various risk and protective factors, including emotional and behavioral difficulties, prosocial behavior, subjective well‐being, EI, self‐esteem, depressive symptoms, empathy, positive and negative affect, and emotional regulation. We hypothesize that measures of suicidal behavior and associated psychological constructs will be positively interconnected. In addition, we expect that in the psychological network, indicators of well‐being and distress will be positively interconnected, with emotional and behavioral problems closely interconnected within their domain and inversely associated across domains.

## 2 Method

### 2.1 Participants

This study used incidental sampling. The sample consisted of a total of 1048 Spanish university students (*M* = 20.5 years; SD = 2.5; 58.2% women) from Miguel Hernández University of Elche, the Catholic University of Murcia, and the University of Alicante. Regarding their academic level, 99.8% were enrolled in undergraduate programs, while 0.2% were pursuing a master's degree. In terms of their year of study, 40.5% were in their first year, 21.1% in the second, 22.3% in the third, 15.1% in the fourth, 0.6% in the fifth, and 0.4% in the sixth year. The research was conducted during the 2018/2019 academic year through a cross‐sectional study carried out online from October to January. The research was conducted during the 2018/2019 academic year through a cross‐sectional study carried out online from October to January. Participants were recruited through incidental sampling. Researchers visited university classrooms in person, explained the purpose of the study, and invited students to participate. The questionnaire was administered online, but students completed it in situ, in their respective classrooms, under the supervision of the researchers to ensure compliance with ethical standards and minimize response biases.

### 2.2 Instruments

Suicidal behavior was assessed using an instrument based on two tools, the *Self‐Injurious Thoughts and Behaviors Interview* (SITBI; Nock et al. 2007) and the *Columbia‐Suicide Severity Rating Scale* (C‐SSRS; Posner et al. 2011). A selection of items from the Spanish versions of the SITBI (García‐Nieto et al. 2013) and the C‐SSRS (Al‐Halabí et al. 2016). The assessment covered five key indicators: death wishes, suicidal ideation, suicide planning, self‐injurious behaviors, and previous suicide attempts. Participants were asked, “Have you ever thought about ending your life?” “Have you ever wished you were dead or that you could fall asleep and never wake up?” “Have you ever thought about how you could take your own life (e.g., taking pills or jumping out of a window) or have you planned how to do it?” “Have you ever deliberately hurt yourself without intending to die?” and “In your lifetime, how many times have you deliberately hurt yourself without intending to die?” Each item assessed both lifetime and past‐year occurrence using a dichotomous response format (yes/no). The selection of these items was based on previous research demonstrating their reliability and validity in assessing suicidal risk among Spanish university students (Blasco et al. 2016).

*The Mental Health Continuum Short Form* (MHC‐SF; Keyes et al. 2008; Spanish version by Aragonés et al. 2011). This measure provides a multidimensional assessment of subjective well‐being: emotional, psychological, and social. It consists of 14 items and six response alternatives that reflect the frequency of experiencing positively formulated symptoms. Although the aforementioned Spanish version was employed, minimal changes were made to ensure it was more faithful to the original, such as using the 6‐point response format and improving the wording of some items, in line with the Spanish version for adolescents validated by Piqueras et al. (2022). The instrument used was validated in a Spanish sample and has shown good psychometric properties (Echeverría et al. 2017).

*Positive and Negative Affect Schedule* (PANAS; Watson et al. 1988). This scale measures recent emotional experiences (past 2 weeks) of the respondents. Based on Furlong et al. (2017), a brief six‐item version of PANAS was used in the present study, which had previously shown high factor loadings according to Crawford and Henry (2004) to measure positive affect (determined, proud, interested) and negative affect (distressed, upset, nervous).

*The Strengths and Difficulties Questionnaire* (SDQ; Goodman 1997). In this study, we used the Spanish version downloaded from the official SDQ website (www.sdqinfo.org) and with permission from www.youthinmind.com to include the SDQ in our electronic survey. It consists, like the original, of 25 questions that evaluate five subscales: emotional symptoms, conduct problems, hyperactivity, peer problems, and prosocial behavior. Strengths are measured through the prosocial behavior subscale, while difficulties are measured through the other four scales. Recently, a version for adults of the SDQ has been developed (using almost identical wording), which has been found to show similar psychometric properties to child/adolescent samples (Brann et al. 2018; Riglin et al. 2024).

*Social Emotional Health Survey—Higher Education and Adults* (SEHS‐HE; Furlong et al. 2017). This instrument is designed for the self‐reported assessment of socio‐emotional competencies. The global covitality measure refers to the outcome of the interaction between all these socio‐emotional competencies and their synergistic effect on mental health. It consists of a total of 36 items, with response options presented on a 6‐point Likert scale, according to the degree of identification with the statements. The use of this measure for the Spanish university population has been supported by a cross‐cultural study with Spanish, Mexican, and Californian populations (Furlong et al. 2021).

*Rosenberg Self‐Esteem Scale* (RSE; Rosenberg 1965). The RSE is a brief self‐report that comprehensively assesses self‐esteem, understood as a positive or negative attitude towards oneself. It classifies individuals into three groups (high, medium, and low self‐esteem) based on 10 items answered on a 4‐point Likert scale.

*The Trait Emotional Intelligence Questionnaire Short Form* (TEIQue‐SF; Petrides 2009; Spanish version by Pérez‐González 2003) is considered one of the most comprehensive, reliable, and valid assessments of global EI as a set of personality dispositions that facilitate emotionally intelligent behavior (Martínez‐Saura et al. 2023). The TEIQue‐SF consists of 30 items and assesses four main factors: well‐being, self‐control, emotionality, and sociability, which can be combined to create a global EI score. In addition, the independent facets of self‐motivation and adaptability are included. Both the main factors and these facets contribute to the overall trait of EI. The TEIQue‐SF scale, along with its Spanish translation, is freely available for academic research purposes and has demonstrated strong psychometric properties (Laborde et al. 2016).

*The Social‐Emotional Distress Survey* (SEDS‐S; Dowdy et al. 2018) is a screening questionnaire that assesses internalizing distress. It consists of 10 items answered on a 4‐point Likert scale. The instrument used was validated in a Spanish sample and has shown good psychometric properties (Rodríguez‐Jiménez et al. 2024).

### 2.3 Procedure

The research was approved by the Clinical Research Ethics Committee (reference: DPS.JPR.03.17). This study was designed as a cross‐sectional, descriptive–correlational study.

The administration of the measurement instruments was conducted online, with 1048 Spanish university students participating during the 2018/2019 academic year. The participants were from Miguel Hernández University of Elche, the Catholic University of Murcia, and the University of Alicante.

The study was presented to the participants as research on emotional well‐being, ensuring the confidentiality of their responses and emphasizing the voluntary nature of their participation. Descriptive statistics and sample scores on the different administered questionnaires were calculated to obtain the participants' profile and the mean scores of the different measures.

### 2.4 Data Analysis

The procedures of Fonseca‐Pedrero et al. (2024) were followed to replicate the design in young people. Descriptive and network analyses were conducted using JASP 0.18.3 and R Studio (2024.04.0+735). For a detailed explanation, see Supporting Information S1: Appendix A.

## 3 Results

We evaluated the prevalence of suicidal behavior indicators in our sample of university students. Overall, 21.5% of participants reported suicidal thoughts, 26.3% indicated that they had wished not to wake up or be dead, 15% considered or formulated specific suicide plans, 5.8% reported at least one suicide attempt, and 18% engaged in NSSI.

### 3.1 Estimated Network of Suicidal Behavior

**FIGURE 1:**
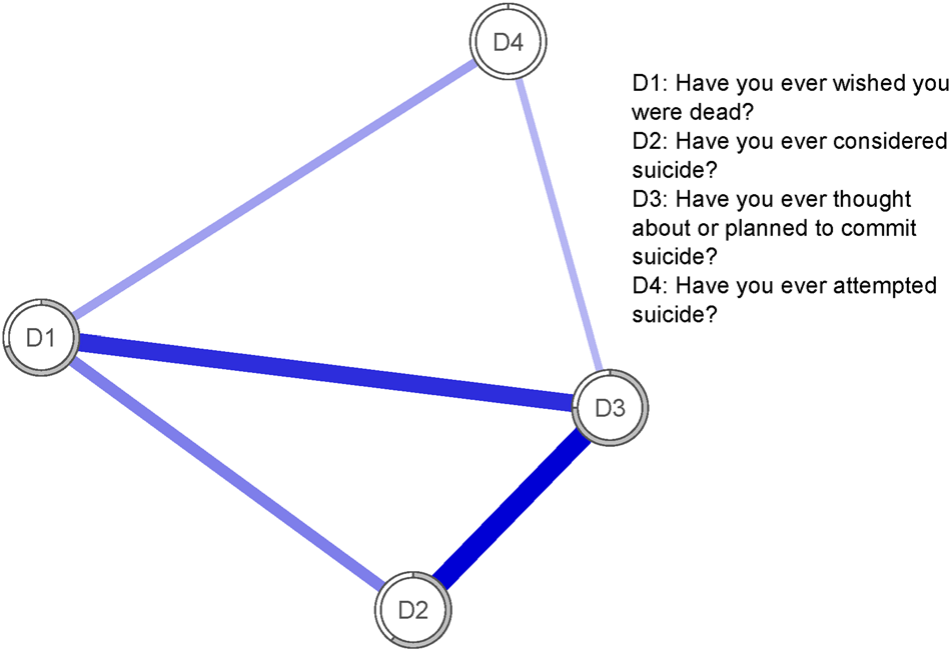
Estimated network of suicidal behavior in young people (*n* = 1048).

Figure 1 shows the estimated network of suicidal behavior in young people.

**FIGURE 2:**
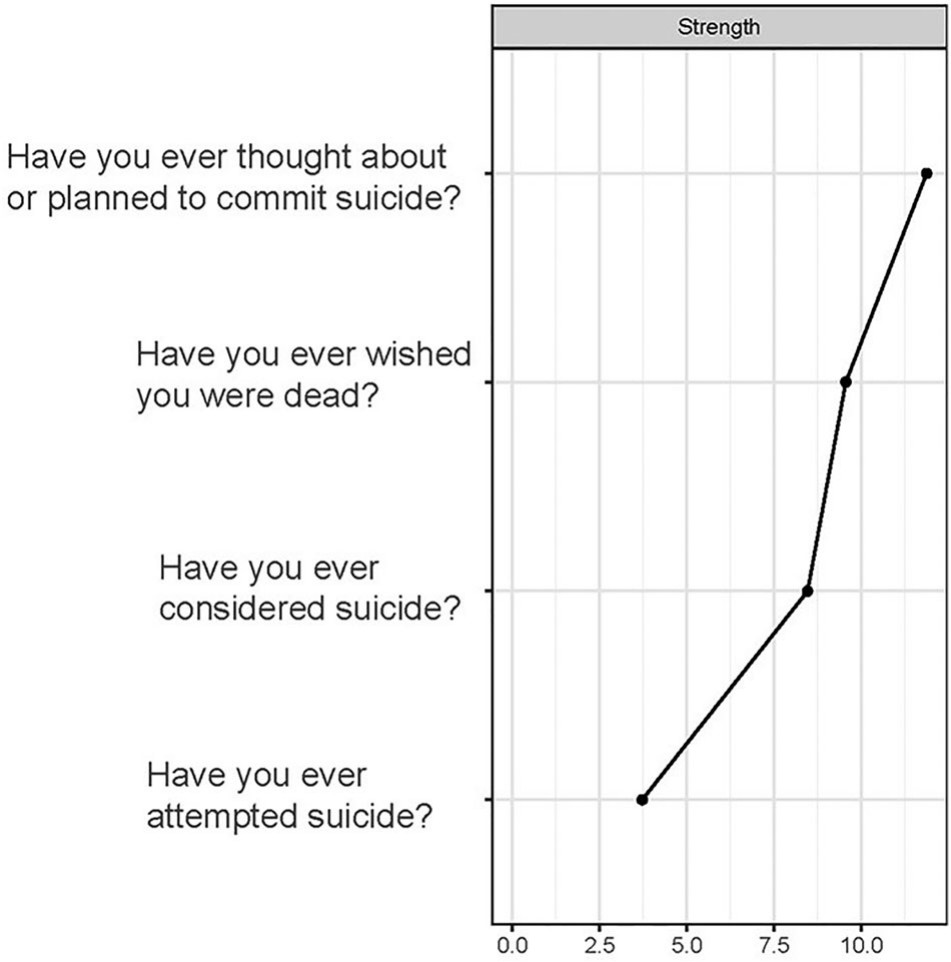
Centrality strength values for the suicidal behavior.

The strongest relationships were found between (D2) “Have you ever considered suicide?” and (D3) “Have you ever thought about or planned to commit suicide?” and between (D3) and (D1) “Have you ever wished you were dead?” The centrality values (Figure 2) showed that the most central node in terms of expected influence was (D3) with the most variance explained (*R* ^2^ = 0.75).

This was followed by D1 with 0.71 and D2 with 0.60. The values of node predictability ranged from 0% to 75%, with an average predictability of 52%. This suggests that in this network on average 52% of the variance in nodes can be explained by their neighbors. It is worth noting that D4 did not explain any percentage of variance in this sample due to its low prevalence).

### 3.2 Estimated Psychological Network of Suicidal Behavior, Risk, and Protective Factors in Young People

**FIGURE 3:**
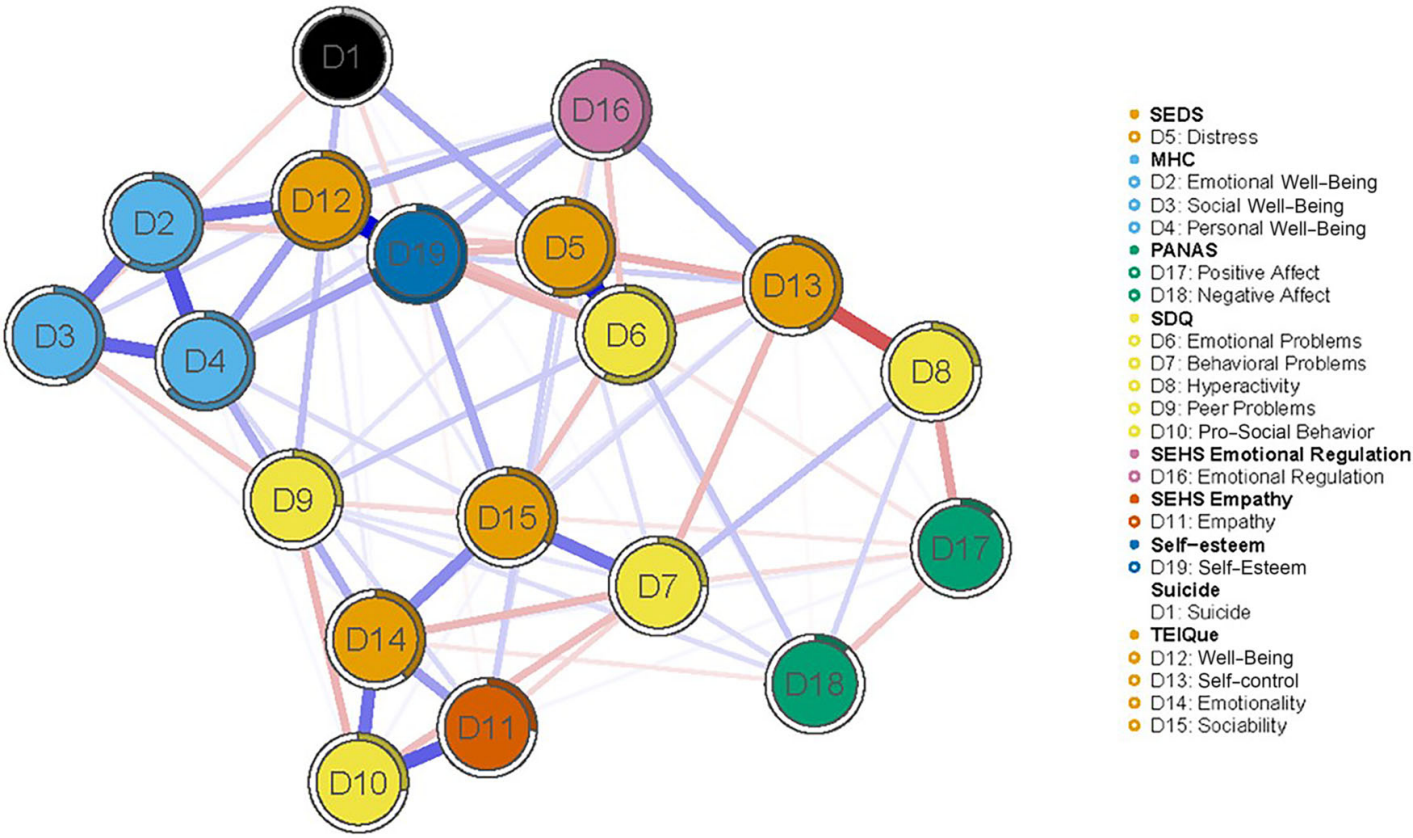
Estimated psychological network of suicidal behavior, risk and protective factors in young people.

**FIGURE 4:**
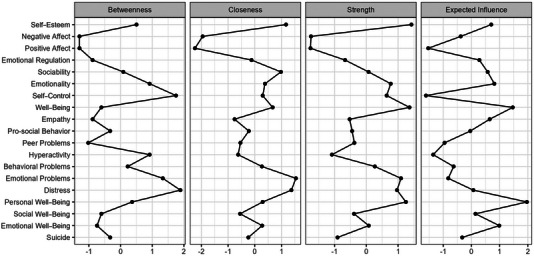
Strength and expected influence indices for the estimated network.

The result of introducing all variables as nodes in the model can be seen in Figure 3 (the weight matrix is shown in Supporting Information S2: Appendix B). The centrality metrics can be found in Figure 4. The most relevant nodes of the network in terms of strength were Self‐Esteem (St = 1.420), Well‐Being (EI, St = 1.360), Personal Well‐Being (St = 1.246), and Emotional Problems (St = 1.092). The least relevant were Positive Affect (St = −1.761) and Negative Affect (St = −1.745). The relationships between the nodes considered protective (e.g., Self‐Esteem and Well‐Being) and risk nodes (e.g., Emotional Problems and Distress) with each other i.e., protective with protective and risk with risk nodes, were strong and significant.

The nodes that activated the network the fastest were Emotional Problems (Clo = 1.539), Distress (Clo = 1.372), Self‐esteem (Clo = 1.169), and Sociability (EI, Clo = 0.979). On the other hand, the nodes that activated the network the slowest were Positive Affect (Clo = −2.240) and Negative Affect (Clo = −1.297). The protective nodes along with the risk nodes were located closer together than close to other nodes (e.g., D14–D12–D1 as protective and D5–D6 as risk factors).

The nodes with the highest connectivity in the network were Distress (Bet = 1.888), Self‐Control (EI, Bet = 1.749), and Emotional Problems (Bet = 1.334). Those with the lowest connectivity were Positive Affect (Bet = −1.297), Negative Affect (Bet = −1.297) and Peer Problems (Bet = −1.020).

Analyzing the indicators as a whole, the proximity, flow speed and strength between nodes D6–D5–D19–D12 indicate that Distress and Emotional Problems would be the main risk factors and Self‐Esteem and Well‐Being (EI) the main protective factors of the network.

In terms of expected influence, the most influential nodes in the network were Personal Well‐Being, Well‐Being (EI) and Emotional Well‐Being (Figure 4). The values of node predictability ranged from 11% to 72%, with an average of 39%. This suggests that in this network on average 39% of the variance in nodes can be explained by the other nodes.

**FIGURE 5:**
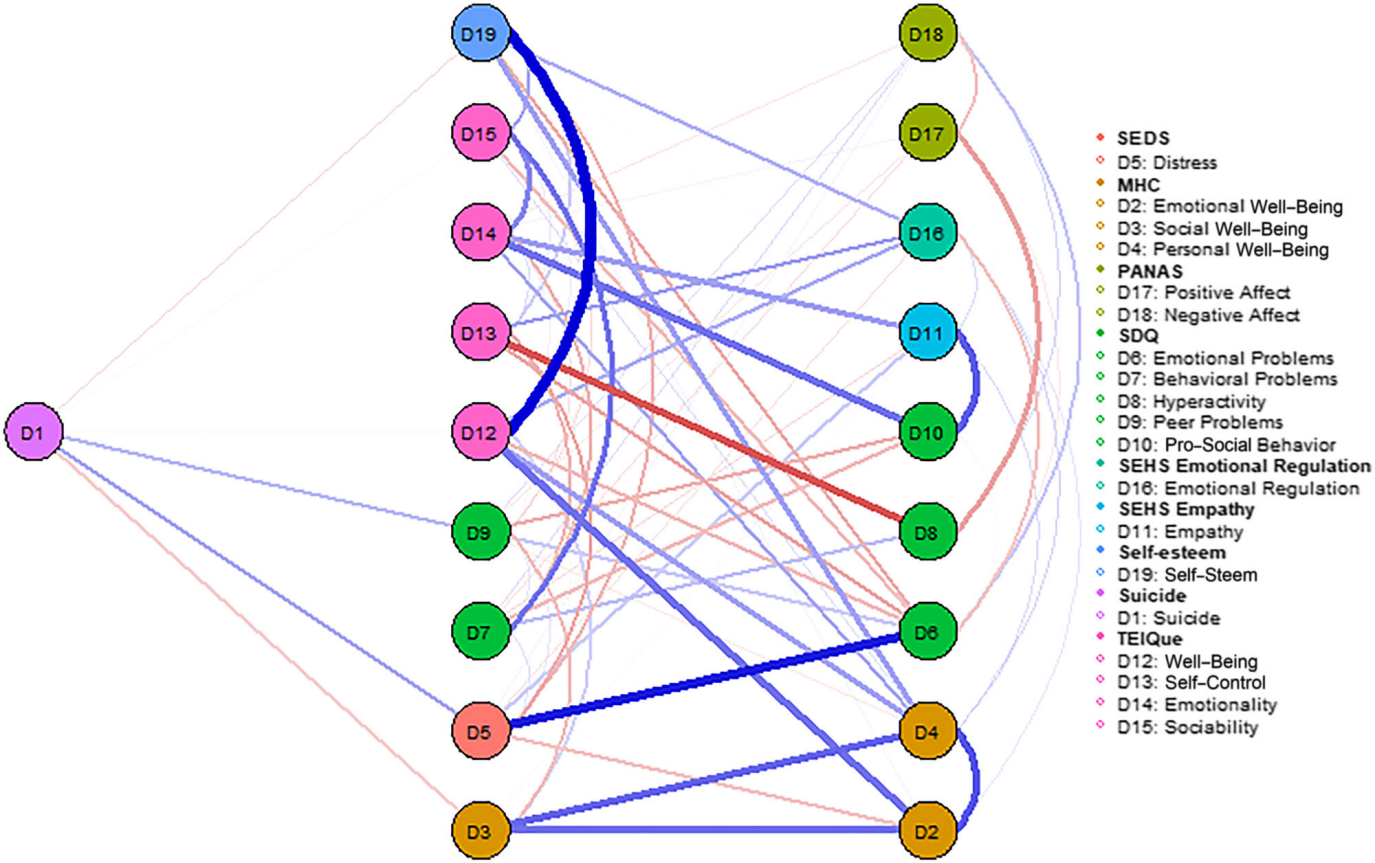
Estimated network's flow diagram.

To facilitate the analysis and interpretation of the specific relationships between the protective and risk nodes of suicidal behavior in young people, a flow chart was developed (Figure 5).

Finally, the stability and accuracy analysis of the network through bootstrap yielded positive results, indicating that the network was accurately estimated (Supporting Information S3: Appendix C).

## 4 Discussion

The primary aim of this study was to analyze the network structure of suicidal behavior in a sample of university students, examining its relationship with various psychological risk and protective factors. This study builds upon the methodological framework established in previous research (Fonseca‐Pedrero et al. 2024), expanding its application to this population. The results reflect significant patterns of interconnection among specific components of suicidal behavior, highlighting the close relationship between the desire to be dead and suicidal planning.

Regarding suicidal behavior, as discussed by Fonseca‐Pedrero et al. (2024), it was observed that the indicators of this phenomenon were strongly connected in the estimated network. The most central nodes were those referring to greater severity, such as considering taking one's own life and previous suicide attempts. Fonseca‐Pedrero et al. (2024) also found that “considering taking one's own life” was the most central node in terms of strength and expected influence. In the estimated network, items related to considering and planning suicide showed particularly strong connections, highlighting “considering taking one's own life” as the central node in terms of strength and expected influence. These findings align with previous research using network analysis to examine suicidal behavior in adults, demonstrating that suicidal ideation and previous attempts form an interconnected network influenced by cognitive, emotional, and social factors (Jones et al. 2021). In line with the network theory of psychopathology proposed by Borsboom (2017), our results suggest that mental disorders can be conceptualized as stable states of a highly connected network, where symptoms reinforce each other. Thus, the severity of suicidal behavior appears to be intrinsically linked to its network structure, reflecting a complex and dynamic interplay of psychological and emotional components.

Regarding risk and protective factors, our findings confirm previous studies identifying depression and negative affect as major risk factors, while self‐esteem and positive affect act as protective factors against suicidal behavior (Kleiman and Nock 2018). Zhou et al. (2023) similarly found that depression was directly associated with both suicidal behavior and NSSI, while social support was negatively correlated with suicidal ideation and NSSI thoughts. Our study further reinforces the centrality of depression in the suicide risk network. However, unlike Zhou et al. (2023), our analysis did not include specific nodes related to NSSI, which may explain certain discrepancies in the observed relationships.

Oliva et al. (2024) identified “Depressed mood” as the most influential central node, followed by anxiety symptoms such as “Feeling nervous,” “Worrying,” “Restless,” and “Trouble relaxing.” In addition, they highlighted a negative connection between “General Health” and “Suicidal thoughts.” In our study, we found that depressive and anxiety symptoms were critical components of the network, with “Depressed mood” as the most central node. The negative connection between general health perception and suicidal ideation observed by Oliva was also reflected in our findings, underscoring the influence of health perception on modulating suicide risk.

On the other hand, previous research has identified “entrapment” as a key psychological construct linked to suicidal behavior, particularly in individuals experiencing a perceived inability to escape from distressing situations (O'Connor and Kirtley 2018). The IMV Model of Suicide (O'Connor 2011) conceptualizes entrapment as a core factor driving suicidal ideation, reinforcing the importance of understanding the dynamic interplay between emotional distress and cognitive factors in the development of suicidal behavior. These findings align with the IMV Model, which emphasizes the transition from defeat and entrapment to suicidal ideation, highlighting the relevance of cognitive and affective components in understanding suicide risk.

Although much of the existing literature focuses on high‐income populations, suicide remains a global public health challenge. In LMICs, research suggests that the prevalence and risk factors for suicidal behavior differ in important ways. For instance, González‐Forteza et al. (2020) reported that suicidal ideation among Mexican adolescents ranges from 10% to 35%, with suicide attempts between 5% and 15%, reflecting significant variation across different socioeconomic contexts. In addition, data from the World Health Organization (WHO 2019) indicate that approximately 79% of all suicides worldwide occur in LMICs, with pesticide ingestion, hanging, and firearms being the most common methods.

These cross‐national differences highlight the importance of cultural, social, and economic factors in shaping suicidal behavior. In LMICs, economic hardship, limited access to mental health services, and social stigma around mental health may contribute to both underreporting and differences in risk profiles (Musyimi et al. 2020). Consequently, prevention strategies need to be culturally adapted to address specific risk factors prevalent in these settings. In this regard, our findings reinforce the importance of adopting a multicomponent approach to suicide prevention, integrating both risk reduction and strength‐based promotion strategies. This aligns with evidence suggesting that interventions targeting psychological resources such as self‐efficacy, emotional regulation, and social connectedness can mitigate the risk of suicidality while fostering resilience (Soto‐Sanz et al. 2019).

However, while these findings are highly relevant for studying suicidal behavior in young adults, it is essential to mention the limitations of this work. Following the classification proposed by Al‐Halabí and Fonseca‐Pedrero (2024), our approach aligns with a universal prevention model, as it seeks to promote well‐being and reduce suicide risk at a broad population level. However, given the strong interconnections observed in the suicidal behavior network, additional selective and indicated strategies should be considered. Advances in ambulatory assessment methodologies enable continuous monitoring of at‐risk individuals, allowing for earlier intervention in cases where rapid shifts in suicidal ideation occur (Fonseca‐Pedrero et al. 2022). Selective prevention could integrate real‐time tracking of psychological states through digital applications, improving crisis response and facilitating timely psychological support (Kleiman et al. 2017). These levels of intervention should be integrated within a comprehensive suicide prevention framework that includes school‐based programs, community screening, and access to evidence‐based psychotherapeutic interventions (Fonseca‐Pedrero et al. 2023).

First, the cross‐sectional nature of the data prevents establishing definitive causal relationships between network components and suicidal behavior over time (Christensen et al. 2014). In addition, the incidental sample may not fully represent the diversity of experiences and contexts in the general population (O'Connor and Nock 2014). Another limitation is that the study population consists solely of university students, limiting the generalizability of our findings. Although labeled as a study on young people, this population represents a specific demographic group with particular stressors and protective factors. More research is needed to validate these findings in non‐university populations, rural communities, and LMICs (O'Connor and Nock 2014). Therefore, although multiple domains of socio‐emotional adjustment have been integrated, it remains crucial to validate these findings in broader and more diverse contexts. In addition, data collection took place during the 2018/2019 academic year, before major global events that have significantly impacted mental health, such as the COVID‐19 pandemic. Given the profound psychosocial effects of the pandemic on young adults, caution is needed when generalizing these findings to the current university student population. Future research should explore whether suicidal behavior patterns and associated psychological constructs have changed in response to these societal shifts.

The findings of this study provide relevant insights for designing comprehensive suicide prevention strategies within a universal prevention framework. Rather than focusing exclusively on reducing depressive symptoms, prevention programs should prioritize strengthening protective psychological resources, such as emotional regulation, self‐efficacy, and social support (Al‐Halabí and Fonseca‐Pedrero 2024). This aligns with the bidimensional conceptualization of mental health, where well‐being and psychopathology are interrelated yet distinct constructs (Greenspoon and Saklofske 2001). Accordingly, interventions in university settings should adopt a holistic perspective that fosters psychosocial strengths, reinforcing resilience against psychological distress (Sufrate‐Sorzano et al. 2022). These results suggest that interventions aimed at improving self‐esteem, promoting EI, and encouraging prosocial behaviors could be effective in reducing vulnerability to suicide among young adults. This is particularly relevant within a universal prevention framework, where fostering psychosocial strengths in the general population can serve as a protective barrier against suicidal behavior. A multilevel prevention strategy is essential for addressing the complexity of suicidal behavior (O'Connor & Pirkis, 2016). Universal prevention should aim to cultivate adaptive coping mechanisms and reinforce positive self‐beliefs to reduce susceptibility to suicidal ideation (WHO, 2021). Selective and indicated interventions, on the other hand, should complement this approach by targeting individuals exhibiting early distress indicators or high‐risk behaviors. For instance, structured screening programs, crisis intervention services, and community‐based monitoring have demonstrated efficacy in reducing suicide risk among vulnerable populations (Fonseca‐Pedrero et al. 2023).

Within a universal prevention framework, personalized strategies can be integrated through flexible and scalable interventions that accommodate diverse student needs. Psychoeducational programs, peer‐led support groups, and digital mental health resources allow for individualized engagement while maintaining a population‐level impact (Al‐Halabí and Fonseca‐Pedrero 2023). This approach aligns with the network theory of mental disorders proposed by Borsboom (2017), which defines mental health as a stable state of a weakly connected network and mental disorders as alternative stable states of a strongly connected network.

Despite the criticisms and limitations associated with network analysis, such as the lack of replicability of centrality indices in some methodological applications (Forbes et al. 2017), these tools remain promising for gaining meaningful insights in various fields of research (Borsboom 2017). The current debate on the replicability and methods used in network estimation underscores the importance of carefully interpreting findings and validating results in diverse contexts and with representative samples. However, even considering these criticisms, these approaches would allow for a more accurate assessment of how risk and protective factors dynamically interact over time, thereby facilitating the development of more effective preventive interventions.

Future studies should continue to explore the dynamic nature of suicidal behavior using methodologies that capture real‐time fluctuations in emotional and cognitive states. Ambulatory assessment, which allows for the momentary and ecological evaluation of psychological processes in natural settings, has shown promise in improving short‐term prediction and understanding of suicidal ideation variability (Kleiman et al. 2017; Fonseca‐Pedrero et al. 2022). The implementation of ambulatory assessment tools could enhance suicide risk detection by identifying shifts in mood, stress levels, and cognitive patterns that precede crisis episodes. Furthermore, integrating ambulatory assessment with multi‐level data (genetic, neurocognitive, and social variables) could provide a more comprehensive understanding of suicide risk trajectories over time.

## 5 Conclusion

In conclusion, this study has provided a detailed and comprehensive view of the network structure of suicidal behavior in young adults, highlighting critical connections between different domains of mental health and emotional well‐being. By integrating the network model, light is shed on the understanding of how factors such as self‐esteem, depression, and empathy interact to influence the risk of suicidal behavior.

These findings underscore the need for longitudinal research that explores the role of protective factors in promoting well‐being and preventing mental health problems, with the aim of advancing towards more personalized and effective intervention strategies in reducing the risk of suicide.

## Author Contributions

**Victoria Soto‐Sanz**: conceptualization, validation, investigation, data curation, writing–original draft. **Álvaro García del Castillo‐López**: methodology, formal analysis, writing–original draft, visualization. **David Pineda**: methodology, formal analysis, visualization. **Raquel Falcó**: conceptualization, validation, investigation, data curation, writing–review and editing. **Tíscar Rodríguez‐Jiménez**: conceptualization, validation, investigation, data curation, writing–review and editing. **Juan C. Marzo**: methodology, formal analysis, resources, writing–review and editing. **José A. Piqueras**: conceptualization, resources, writing–review and editing, supervision.

## Conflicts of Interest

The authors declare no conflicts of interest.

### Peer Review

The peer review history for this article is available at https://publons.com/publon/10.1002/brb3.70457

## Supporting information



Supporting Information

Supporting Information

Supporting Information

## Data Availability

The data that support the findings of this study are available upon reasonable request.
