# Supplementary material for: Suicidal Behavior in University Students in Spain: a Network Analysis
Source: Brain Behav. 2025 Apr 18;15(4):e70457. doi: 10.1002/brb3.70457 (PMC12006927; doi:10.1002/brb3.70457)
Supplement: Supplementary file 1 — Supporting Information [file BRB3-15-e70457-s002.docx]

APPENDIX A

*Network estimation*

We first estimated suicidal behavior using using an instrument based on two tools, *the Self-Injurious Thoughts and Behaviors Interview* SITBI (Nock et al., 2007) and the *Columbia-Suicide Severity Rating Scale* (C-SSRS; Posner et al., 2011). A selection of items from the Spanish versions of the SITBI (García-Nieto et al., 2013) and the C-SSRS (Al-Halabí et al., 2016) were administered to evaluate 5 indicators of the suicidal spectrum in a self-report format, which has proven to be accurate for Spanish university students (Blasco et al., 2016)., obtaining a general attitude evaluation towards suicide as a function of motivation, desire, cognitions and intention. Second, the psychological structure of suicidal behavior was estimated using total scores for suicidal behavior, self-esteem, emotional regulation, emotional and behavioral difficulties, empathy, positive and negative affect, well-being, distress, and emotional intelligence.

Network analysis was used to analyze the links between the variables. In this type of analysis, the variables are called 'nodes' and the relationships between them are called 'edges'. The nodes are represented by a circumference and the edges by lines connecting them. The thickness of the line indicates the strength of the relationship, with blue indicating a positive relationship and red indicating a negative relationship. This type of analysis is increasingly used in the psychological and social sciences because it provides a clear visual representation of the relationships between variables and helps to better explain the causal interactions between them. To analyze cross-sectional data, the Gaussian Graphical Model (GGM) is commonly used for continuous data, while the Ising model is preferred for binary data. In this sense, GGM was used to construct the network model using the variables described in the previous section as network nodes and the Ising Model was used for the dichotomous variables through the 'IsingFit’ package. The network was designed using the Fruchterman-Reingold algorithm to place the most relevant nodes of the network in the center, while relegating the weakest nodes to the extremes. To reduce the number of weak edges, the sparse inverse covariance matrix was estimated with a lasso penalty (L1) using the 'glasso' package which limits over-fitting and leads to more parsimonious and reproducible models (Epskamp & Fried, 2018). The EBICglasso algorithm was used to calculate a regularized GGM from a correlation matrix input and integrating the graphical lasso algorithm (Isvoranu & Epskamp, 2023). The Tuning parameter, which controls the degree of sparsity, was set to .25 to create a structure that minimized spurious edges (Fonseca-Pedrero et al., 2024). To visualize the data and for the network estimation, package 'qgraph’ was used (Epskamp et al., 2012). A nonparametric bootstrap with 1,000 samples was employed to evaluate the accuracy of edge weights through the ‘bootnet’ package. The syntax used can be viewed in detail at OSF link.

*Network inference*

To determine the most relevant nodes in the model, the measures of strength, betweenness, closeness, expected influence and predictability were calculated.

Strength measures the number of direct connections a node has. A node has a strong centrality the more connections it has with other nodes, although more is not necessarily better, so it is therefore recommended to consult other measures such as betweenness centrality (Barrat et al., 2004).

Betweenness measures how often a node acts as a bridge on the shortest paths between other nodes. We will therefore understand that a node has a higher betweenness centrality the more short paths between two nodes pass through the node in question, characterising this node as fundamental to the functioning of the network. (Hansen et al., 2020).

Closeness centrality measures how close a node is to others in the network, using the total length of the shortest paths. While node strength examines the extent of a node's direct connections with other nodes in the network, closeness explores the level of its indirect connections, and can be understood as the expected rate at which things flow through the network (Borgatti, 2005).

Expected influence calculates the expected influence of a node based on its direct and indirect connectivity, integrating the concepts of strength and structure. This metric considers directionality by distinguishing between positive and negative edge weights and avoids using absolute values when calculating node strength, instead favoring actual values (Robinaugh et al., 2016).

Finally, as a complement to the centrality measures, the predictability of the nodes was calculated. This value indicates what proportion of the variance of each node is explained by the model, so the closer the value is to 1, the better the model predicts the node (Haslbeck & Fried, 2017).

*Network stability and accuracy*

To ensure that the network estimates were sufficiently stable and accurate, bootstrap techniques were used. The correlation stability coefficient (CS-coefficient) indicates the proportion of cases that can be removed while still maintaining a correlation of over 0.7 between the resulting centrality estimate and the original estimate in 95% of case-dropping bootstrap resamples (Papageorgiou et al., 2019). Following the recommendation of previous studies, cut-off values of .25 were established as sufficient and .50 as good (Epskamp et al., 2018).
