# Supplementary material for: Suicidal Behavior in University Students in Spain: a Network Analysis
Source: Brain Behav. 2025 Apr 18;15(4):e70457. doi: 10.1002/brb3.70457 (PMC12006927; doi:10.1002/brb3.70457)
Supplement: Supplementary file 2 — Supporting Information [file BRB3-15-e70457-s003.docx]

APPENDIX B

Table A1. Estimated network weights matrix

|  | | **Network** | | | | | | | | | | | | | | | | | | | | | | | | | | | | | | | | | | | | | |
| --- | --- | --- | --- | --- | --- | --- | --- | --- | --- | --- | --- | --- | --- | --- | --- | --- | --- | --- | --- | --- | --- | --- | --- | --- | --- | --- | --- | --- | --- | --- | --- | --- | --- | --- | --- | --- | --- | --- | --- |
| **Variable** | | **Self-steem** | | **Well-being** | | **Emotional Well-being** | | **Emotionality** | | **Personal Well-being** | | **Emotional Problems** | | **Suicide** | | **Social Well-being** | | **Distress** | | **Behavioral Problems** | | **Hyperactivity** | | **Peer Problems** | | **Pro-social Behavior** | | **Empathy** | | **Self-control** | | **Sociability** | | **Emotional Regulation** | | **Positive Affect** | | **Negative Affect** | |
| Self-steem |  | 0.000 |  | 0.413 |  | 0.020 |  | 0.000 |  | 0.148 |  | -0.119 |  | -0.068 |  | 0.000 |  | -0.108 |  | 0.000 |  | 0.000 |  | 0.000 |  | 0.000 |  | -0.021 |  | 0.073 |  | 0.135 |  | 0.115 |  | 0.000 |  | 0.000 |  |
| Well-being |  | 0.413 |  | 0.000 |  | 0.229 |  | 0.030 |  | 0.148 |  | -0.104 |  | -0.011 |  | 0.066 |  | -0.019 |  | 0.000 |  | 0.000 |  | -0.019 |  | 0.000 |  | 0.000 |  | 0.010 |  | 0.032 |  | 0.124 |  | 0.000 |  | 0.000 |  |
| Emotional Well-being |  | 0.020 |  | 0.229 |  | 0.000 |  | 0.000 |  | 0.273 |  | 0.000 |  | 0.000 |  | 0.229 |  | -0.090 |  | 0.000 |  | 0.000 |  | 0.000 |  | 0.000 |  | 0.000 |  | 0.000 |  | 0.000 |  | 0.042 |  | 0.000 |  | 0.000 |  |
| Emotionality |  | 0.000 |  | 0.030 |  | 0.000 |  | 0.000 |  | 0.129 |  | 0.000 |  | 0.035 |  | 0.000 |  | 0.000 |  | -0.115 |  | 0.000 |  | -0.056 |  | 0.227 |  | 0.153 |  | 0.058 |  | 0.188 |  | 0.000 |  | 0.024 |  | -0.041 |  |
| Personal Well-being |  | 0.148 |  | 0.148 |  | 0.273 |  | 0.129 |  | 0.000 |  | 0.000 |  | 0.000 |  | 0.245 |  | 0.000 |  | 0.000 |  | 0.000 |  | -0.037 |  | 0.013 |  | 0.059 |  | 0.000 |  | 0.057 |  | 0.068 |  | 0.000 |  | 0.000 |  |
| Emotional Problems |  | -0.119 |  | -0.104 |  | 0.000 |  | 0.000 |  | 0.000 |  | 0.000 |  | 0.000 |  | 0.000 |  | 0.367 |  | 0.000 |  | 0.000 |  | 0.080 |  | 0.019 |  | 0.000 |  | -0.131 |  | -0.098 |  | -0.091 |  | -0.041 |  | 0.086 |  |
| Suicide |  | -0.068 |  | -0.011 |  | 0.000 |  | 0.035 |  | 0.000 |  | 0.000 |  | 0.000 |  | -0.112 |  | 0.169 |  | 0.021 |  | 0.000 |  | 0.141 |  | 0.000 |  | 0.000 |  | -0.043 |  | 0.037 |  | 0.000 |  | 0.000 |  | 0.000 |  |
| Social Well-being |  | 0.000 |  | 0.066 |  | 0.229 |  | 0.000 |  | 0.245 |  | 0.000 |  | -0.112 |  | 0.000 |  | 0.000 |  | 0.000 |  | 0.004 |  | -0.091 |  | 0.000 |  | 0.020 |  | 0.000 |  | 0.000 |  | 0.000 |  | 0.000 |  | 0.000 |  |
| Distress |  | -0.108 |  | -0.019 |  | -0.090 |  | 0.000 |  | 0.000 |  | 0.367 |  | 0.169 |  | 0.000 |  | 0.000 |  | 0.048 |  | 0.000 |  | 0.029 |  | 0.000 |  | 0.080 |  | -0.124 |  | 0.000 |  | -0.049 |  | 0.000 |  | 0.025 |  |
| Behavioral Problems |  | 0.000 |  | 0.000 |  | 0.000 |  | -0.115 |  | 0.000 |  | 0.000 |  | 0.021 |  | 0.000 |  | 0.048 |  | 0.000 |  | 0.099 |  | 0.040 |  | -0.103 |  | -0.062 |  | -0.110 |  | 0.222 |  | 0.000 |  | -0.053 |  | 0.057 |  |
| Hyperactivity |  | 0.000 |  | 0.000 |  | 0.000 |  | 0.000 |  | 0.000 |  | 0.000 |  | 0.000 |  | 0.004 |  | 0.000 |  | 0.099 |  | 0.000 |  | 0.000 |  | 0.000 |  | 0.000 |  | -0.274 |  | 0.000 |  | 0.000 |  | -0.148 |  | 0.067 |  |
| Peer Problems |  | 0.000 |  | -0.019 |  | 0.000 |  | -0.056 |  | -0.037 |  | 0.080 |  | 0.141 |  | -0.091 |  | 0.029 |  | 0.040 |  | 0.000 |  | 0.000 |  | -0.120 |  | 0.000 |  | 0.000 |  | -0.065 |  | 0.000 |  | -0.040 |  | 0.052 |  |
| Pro-social Behavior |  | 0.000 |  | 0.000 |  | 0.000 |  | 0.227 |  | 0.013 |  | 0.019 |  | 0.000 |  | 0.000 |  | 0.000 |  | -0.103 |  | 0.000 |  | -0.120 |  | 0.000 |  | 0.259 |  | 0.000 |  | -0.010 |  | 0.000 |  | 0.000 |  | 0.000 |  |
| Empathy |  | -0.021 |  | 0.000 |  | 0.000 |  | 0.153 |  | 0.059 |  | 0.000 |  | 0.000 |  | 0.020 |  | 0.080 |  | -0.062 |  | 0.000 |  | 0.000 |  | 0.259 |  | 0.000 |  | 0.000 |  | 0.000 |  | 0.062 |  | 0.016 |  | 0.000 |  |
| Self-control |  | 0.073 |  | 0.010 |  | 0.000 |  | 0.058 |  | 0.000 |  | -0.131 |  | -0.043 |  | 0.000 |  | -0.124 |  | -0.110 |  | -0.274 |  | 0.000 |  | 0.000 |  | 0.000 |  | 0.000 |  | 0.039 |  | 0.145 |  | 0.010 |  | -0.009 |  |
| Sociability |  | 0.135 |  | 0.032 |  | 0.000 |  | 0.188 |  | 0.057 |  | -0.098 |  | 0.037 |  | 0.000 |  | 0.000 |  | 0.222 |  | 0.000 |  | -0.065 |  | -0.010 |  | 0.000 |  | 0.039 |  | 0.000 |  | 0.000 |  | -3.457×10^-5^ |  | 0.000 |  |
| Emotional Regulation |  | 0.115 |  | 0.124 |  | 0.042 |  | 0.000 |  | 0.068 |  | -0.091 |  | 0.000 |  | 0.000 |  | -0.049 |  | 0.000 |  | 0.000 |  | 0.000 |  | 0.000 |  | 0.062 |  | 0.145 |  | 0.000 |  | 0.000 |  | 0.000 |  | 0.000 |  |
| Positive Affect |  | 0.000 |  | 0.000 |  | 0.000 |  | 0.024 |  | 0.000 |  | -0.041 |  | 0.000 |  | 0.000 |  | 0.000 |  | -0.053 |  | -0.148 |  | -0.040 |  | 0.000 |  | 0.016 |  | 0.010 |  | -3.457×10^-5^ |  | 0.000 |  | 0.000 |  | -0.091 |  |
| Negative Affect |  | 0.000 |  | 0.000 |  | 0.000 |  | -0.041 |  | 0.000 |  | 0.086 |  | 0.000 |  | 0.000 |  | 0.025 |  | 0.057 |  | 0.067 |  | 0.052 |  | 0.000 |  | 0.000 |  | -0.009 |  | 0.000 |  | 0.000 |  | -0.091 |  | 0.000 |  |
|  | | | | | | | | | | | | | | | | | | | | | | | | | | | | | | | | | | | | | | | |
