# Supplementary material for: Suicidal Behavior in University Students in Spain: a Network Analysis
Source: Brain Behav. 2025 Apr 18;15(4):e70457. doi: 10.1002/brb3.70457 (PMC12006927; doi:10.1002/brb3.70457)
Supplement: Supplementary file 3 — Supporting Information [file BRB3-15-e70457-s001.docx]

APPENDIX C


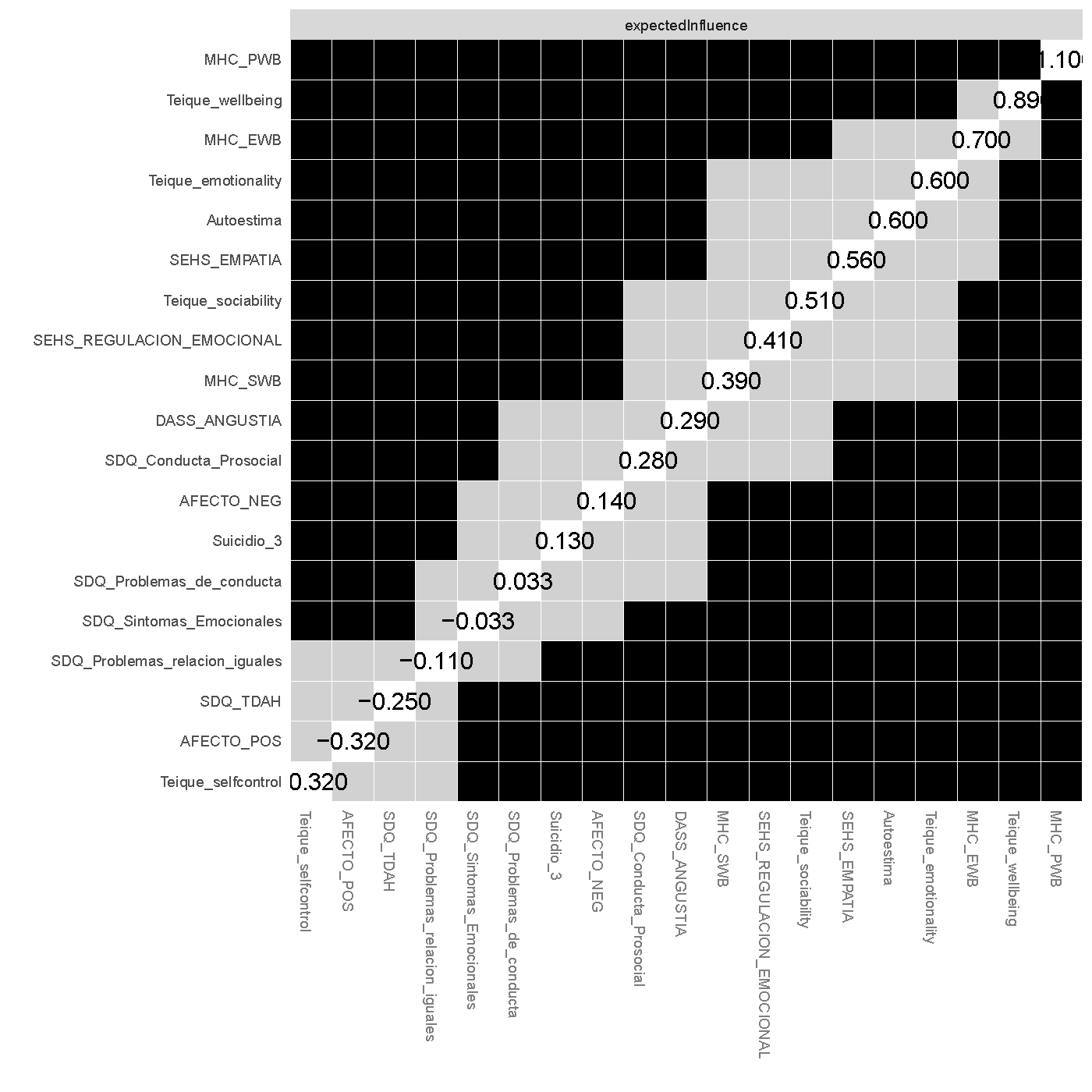
Figure B1. Plot centrality stability


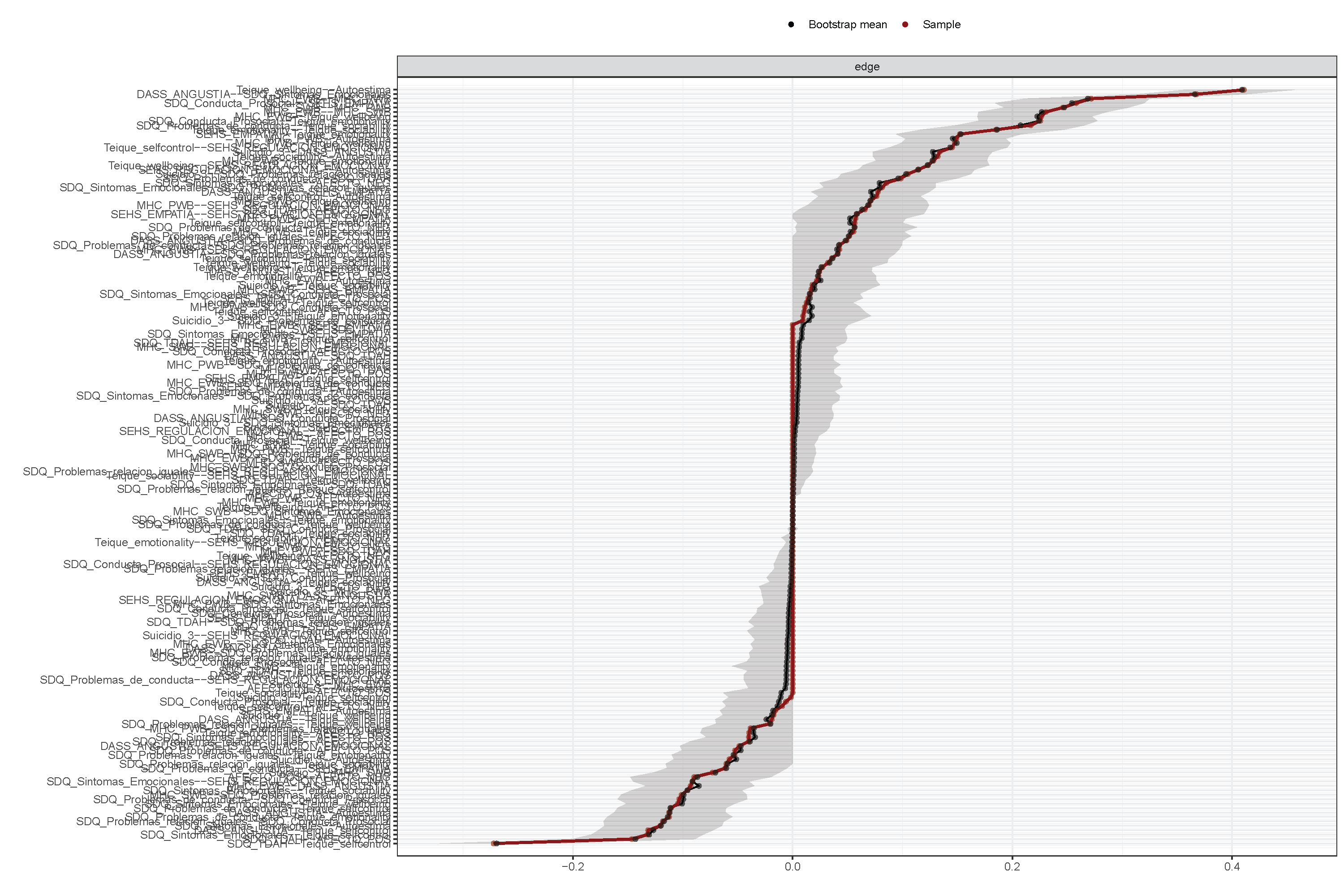


Figure B2. Estimation accuracy of the weighted-edges (red line) and the 95% confidential interval of the estimates (gray line) for the network


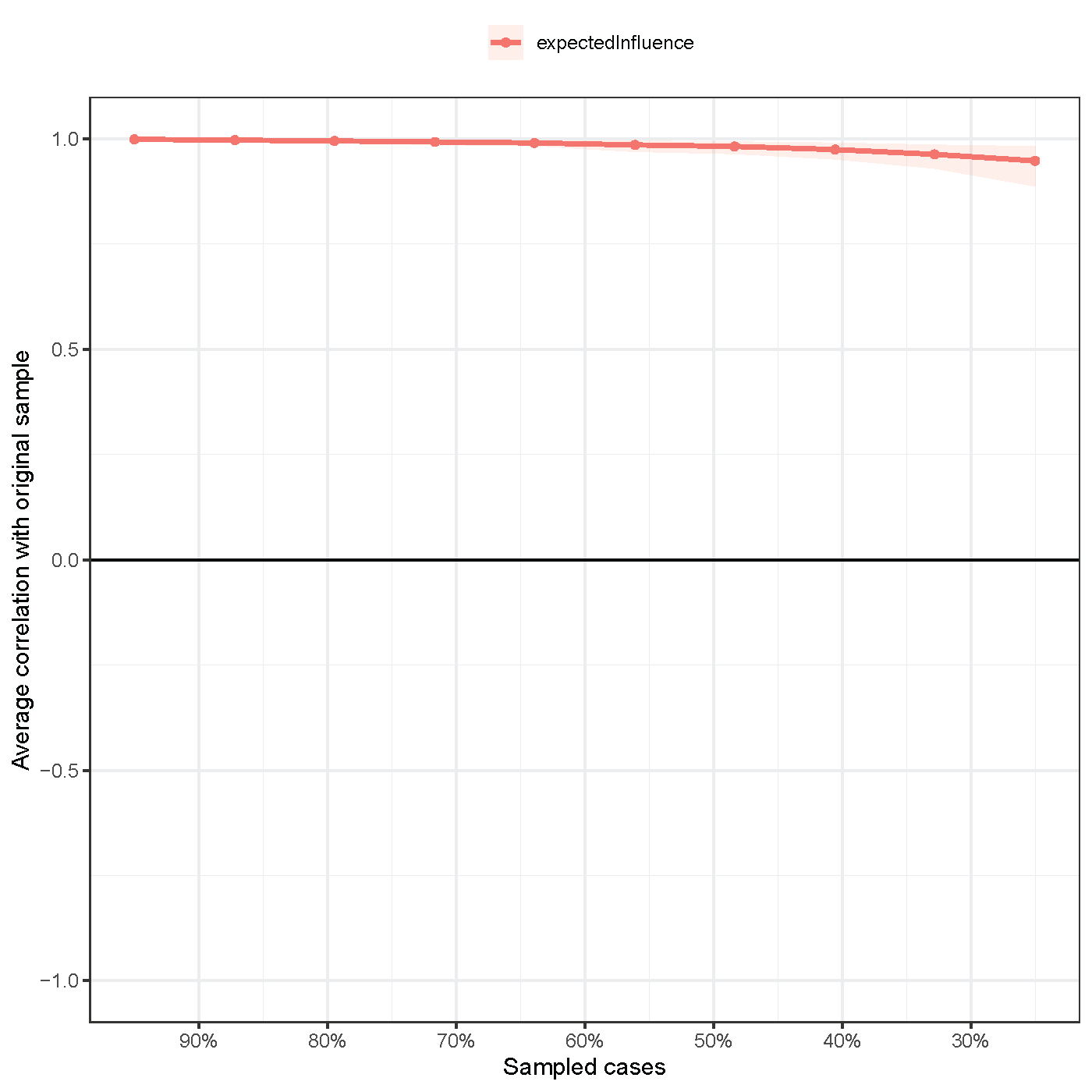


Figure B3. Stability of the centrality indices for the network
